# Caloric restriction favorably impacts metabolic and immune/inflammatory profiles in obese mice but curcumin/piperine consumption adds no further benefit

**DOI:** 10.1186/1743-7075-10-29

**Published:** 2013-03-27

**Authors:** Junpeng Wang, Sally M Vanegas, Xiaogang Du, Timothy Noble, Jean-Marc A Zingg, Mohsen Meydani, Simin Nikbin Meydani, Dayong Wu

**Affiliations:** 1 Nutritional Immunology Laboratory, Jean Mayer USDA Human Nutrition Research Center on Aging at Tufts University, 711 Washington Street, Boston, MA 02111, USA; 2 Vascular Biology Laboratory, Jean Mayer USDA Human Nutrition Research Center on Aging at Tufts University, Boston, MA, 02111, USA; 3 Department of Pathology, Sackler Graduate School of Biomedical Science, Tufts University, Boston, MA, 02111, USA

**Keywords:** Obesity, Caloric restriction, Inflammation, T cells, Curcumin, Piperine

## Abstract

**Background:**

Obesity is associated with low-grade inflammation and impaired immune response. Caloric restriction (CR) has been shown to inhibit inflammatory response and enhance cell-mediated immune function. Curcumin, the bioactive phenolic component of turmeric spice, is proposed to have anti-obesity and anti-inflammation properties while piperine, another bioactive phenolic compound present in pepper spice, can enhance the bioavailability and efficacy of curcumin. This study sought to determine if curcumin could potentiate CR’s beneficial effect on immune and inflammatory responses in obesity developed in mice by feeding high-fat diet (HFD).

**Methods:**

Mice were fed a HFD for 22 wk and then randomized into 5 groups: one group remained on HFD *ad libitum* and the remaining 4 groups were fed a 10% CR (reduced intake of HFD by 10% but maintaining the same levels of micronutrients) in the presence or absence of curcumin and/or piperine for 5 wk, after which CR was increased to 20% for an additional 33 wk. At the end of the study, mice were sacrificed, and spleen cells were isolated. Cells were stimulated with T cell mitogens, anti-CD3/CD28 antibodies, or lipopolysaccharide to determine T cell proliferation, cytokine production, and CD4^+^ T cell subpopulations.

**Results:**

Compared to HFD control group, all CR mice, regardless of the presence of curcumin and/or piperine, had lower body weight and fat mass, lower levels of blood glucose and insulin, and fewer total spleen cells but a higher percentage of CD4^+^ T cells. Additionally, they demonstrated lower production of pro-inflammatory cytokines IL-1β and TNF-α, a trend toward lower IL-6, and lower production of PGE_2_, a lipid molecule with pro-inflammatory and T cell-suppressive properties. Mice with CR alone had higher splenocyte proliferation and IL-2 production, but this effect of CR was diminished by spice supplementation. CR alone or in combination with spice supplementation had no effect on production of cytokines IL-4, IL-10, IFN-γ, and IL-17, or the proportion of different CD4^+^ T cell subsets.

**Conclusion:**

CR on an HFD favorably impacts both metabolic and immune/inflammatory profiles; however, the presence of curcumin and/or piperine does not amplify CR’s beneficial effects.

## Introduction

Excessive energy intake results in weight gain leading to obesity [1]. Obesity, which is increasing worldwide, is a well-recognized risk factor for several chronic diseases including diabetes, cardiovascular diseases, metabolic syndrome, and several forms of cancer [2]. Obesity-associated changes in number, distribution, and function of immune cells are believed to be among the key contributors to the pathogenesis of these diseases. Furthermore, obesity has been shown to be associated with an increased susceptibility to infection [3,4]. Various strategies focused on energy reduction or increasing energy expenditures have been developed. However, there is clearly a lack of effectiveness in most of these approaches as indicated by the fact that the prevalence of obesity is escalating. Thus, identifying food patterns or dietary components with characteristics capable of accelerating weight loss in overweight individuals undergoing a weight reduction or caloric restriction (CR) regimen would be of great value and significance.

CR has been shown to modulate energy balance and extend the survival and maximal lifespan in experimental animal models [5,6]. A limited number of studies have demonstrated that CR favorably impacts immune system functions including both innate and acquired immunities [7-10]. Studies have also suggested that certain dietary components, such as spices and their bioactive components, may exert a beneficial effect on obesity and related metabolic disorders. Among these spices, curcumin, the major bioactive polyphenol present in the spice turmeric, has been shown to possess antioxidant, anticancer, anti-angiogenesis, anti-obesity and chemotherapeutic properties [11]. We and others have reported that dietary curcumin supplementation reduces adiposity and weight gain in a mouse model of high fat diet-induced obesity [12,13]. Curcumin is also known to affect several aspects of immune function including inhibition of T cell proliferation, dendritic cell maturation, and production of pro-inflammatory cytokines IL-6, TNF-α, and IL-17 [14]. However, curcumin’s bioavailability is limited after its ingestion. Piperine, a main component present in pepper, has been reported not only to increase absorption and bioavailability of curcumin by more than 100% [15], but also to demonstrate in and of itself anti-inflammatory [16], anti-tumor [17], and immuno-modulatory [17-19] activities.

In rodents, high fat diet-induced obesity models are the best parallels for human obesity [20]. Obese mice infected with influenza virus have a higher mortality associated with a significant reduction of NK cell cytotoxicity accompanied by a diminished release of IFN-α and IFN-β, suggesting impaired immune function during obesity [3]. Recent studies reported that obesity could also result in a change in CD4^+^ T cell subsets, including pro-inflammatory Th1 and Th17 cells and anti-inflammatory regulatory T cells (Treg) in adipose or immune-associated tissues [21-23]. However, little is known about the impact of CR on CD4^+^ T cell subpopulations, particularly in the obese host. Further, it would be interesting to determine whether the beneficial effect of CR on HFD-induced obesity might be further potentiated by concurrent supplementation with dietary spice curcumin, with or without addition of piperine, to enhance bioavailability and/or efficacy of curcumin. Therefore, we conducted the current study to address these gaps in knowledge.

## Materials and methods

### Animals and diets

Eight-wk old male C57BL/6 mice (Jackson Laboratory) were fed a Western style high fat diet (Harlan Teklad, formulation #TD 06433, 44% calorie from fat) for 22 wk. Mice were then divided into 5 groups (9–10 mice/group): 1) control (mice continuing on HFD *ad libitum*), 2) CR (HFD intake reduced by 10% for 5 wk and then by 20% for another 33 weeks), 3) CR + curcumin (1 g/kg diet), 4) CR + curcumin (1 g/kg) + piperine (50 mg/kg), and 5) CR + piperine (50 mg/kg). CR diets were adjusted to provide the same intake levels for total protein, minerals, and vitamins as in the control HF diet. We intentionally designed the study to mimic the situation in which obese adults adopt a long term, healthy regimen from a given point onward to see whether obesity-associated adverse consequences can be prevented or diminished. The mice were housed individually in shoebox polycarbonate cages under 12 h light/dark cycles. Water was provided *ad-libitum,* and mice were weighed weekly. At the end of the study, mice were killed with CO_2_ asphyxiation followed by exsanguination. Tissues were collected post-mortem. All conditions and handling of the animals were approved by the Animal Care and Use Committee of the Jean Mayer USDA Human Nutrition Research Center on Aging at Tufts University and conducted according to the NIH Guidelines for the Care and Use of Laboratory Animals.

### Total body fat

Total fat mass was measured using a magnetic resonance imaging (MRI) machine, EchoMRI-100 (EchoMRI, Houston, TX). Non-anesthetized mice were placed in a restraining tube, which is inserted into the chamber unit of the MRI machine, and then their body composition (fat mass, lean mass, free water, and total body water) was determined.

### Plasma glucose and insulin

After mice were euthanized, plasma was collected and stored at -80°C. Glucose and insulin concentrations in the plasma were measured using Glucose Colorimetric Assay Kit (Cayman Chemical Company, Ann Arbor, MI) and Ultra Sensitive Mouse Insulin ELISA Kit (Crystal Chem Inc., Downers Grove, IL), respectively, following the kit instructions.

### Splenocyte proliferation

After mice were euthanized, the spleens were aseptically removed, and single-cell suspensions were prepared. Cells were seeded into 96-well round-bottom cell culture plates (2 × 10^5^/well) and incubated in the presence of Con A (Sigma-Aldrich, St. Louis, MO) at 0.5, 1.5, or 3 μg/ml, PHA (Difco Laboratories, Detroit, MI) at 2.5, 5, or 20 μg/ml, or plate-coated anti-CD3 (5 μg/ml) plus soluble anti-CD28 (1 μg/ml) (anti-CD3/CD28, BD Pharmingen, San Diego, CA) for 72 h. Cultures were pulsed with [^3^H]-thymidine (1 μCi/well, PerkinElmer Life Sciences, Boston, MA) during the last 4 h. Cells were harvested by a MicroBeta FilterMate Universal Harvester (PerkinElmer), and cell proliferation was quantified as the amount of [^3^H]-thymidine incorporated into DNA as determined by liquid-scintillation counting in a MicroBeta2 Plate Counter (PerkinElmer). Data are expressed as counts per minute (cpm).

### Cytokine production

Splenocytes in 24-well culture plates (3 × 10^6^/well) were cultured in the presence of lipopolysaccharide (LPS) (1 μg/ml) for 24 h for production of inflammatory cytokines IL-1β, IL-6, and TNF-α, and lipid inflammatory mediator prostaglandin E_2_ (PGE_2_), or in the presence of Con A (1.5 μg/ml) or plate-coated anti-CD3 (5 μg/ml) plus soluble anti-CD28 (1 μg/ml) for 48 h for production of T cell cytokines IFN-γ, IL-2, IL-4, and IL-10. The supernatants were collected and measured using ELISA for IL-1β (kit from R&D system, Minneapolis, MN), IL-6, TNFα, IFN-γ, IL-2, IL-4, and IL-10 (all from BD Pharmingen). PGE_2_ was measured using PGE_2_ kit from Meso Scale Discovery (MSD, Gaithersburg, MD) and analyzed using MSD platform.

### Flow cytometry

To determine the percentage of Treg cells, splenocytes were blocked using anti-CD16/32 (Fc block, BD Pharmingen) and then multi-stained with fluorescence-conjugated anti-CD4, anti-CD25, and anti-Foxp3 using the mouse Foxp3 Buffer Set (all from BD Pharmingen). To determine cytokine production, after splenocytes were stimulated with 50 ng/ml PMA and 500 ng/ml ionomycin (both from Sigma-Aldrich) in the presence of monensin (BD Pharmingen) for 4 h, the cells were blocked, fixed and permeabilized with Cytofix/Cytoperm kit (BD Pharmingen), and stained with fluorescence-conjugated antibodies. The antibodies used for flow cytometry were as follows: anti-CD4, anti-IL-2, anti-IFN-γ, and anti-IL-10 from eBiosciences, and anti-IL-4 and anti-IL-17 from BD Pharmingen. Cytometric measurements were conducted using an Accuri C6 flow cytometer, and acquired data were analyzed with FlowJo7.6 software (Treestar Inc., Ashland, OR).

### Statistical analysis

All results were expressed as means ± SEM. Statistical analysis was conducted using Systat 12 statistical software. Differences were determined using 1-way ANOVA followed by Tukey’s HSD post hoc procedure. Significance was set at P < 0.05.

## Results

### Effect of CR, curcumin, and piperine on body weight and total body fat

**Figure 1:**
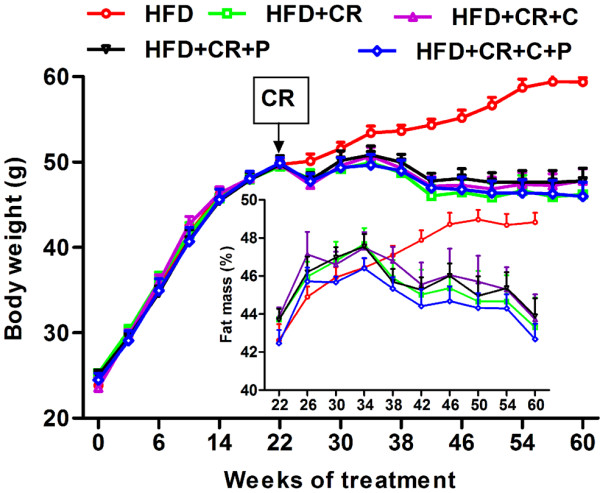
**Effect of CR and spice compounds on body weight and fat mass. **Mice were fed experimental diets for 60 wk as described in the “Materials and methods” section. Body weight and fat mass were recorded at the time points as indicated. Values are means ± SEM, n = 9-10 mice/group. HFD: high fat diet, CR: caloric restriction, C: curcumin, P: piperine.

During the 22-wk of HFD feeding and prior to starting CR and spice compound supplementation, mice in all groups gained weight steadily. After the switching point at wk 22, the mice in HFD control group continued to gain weight, plateauing at wk 57 (3 wk before the end of study), while the mice in all CR groups, with or without curcumin or piperine supplementation, showed a similar weight loss pattern: their weights fluctuated early on, then dropped and stabilized, and eventually ended with a significantly lower average weight compared to HFD control group (Figure 1) by 17-19% (P < 0.001). Similar to the changes in body weight, although with some delay in time, all of the mice in the CR groups had less fat mass compared to the HFD control group, regardless of the presence of curcumin/piperine (Figure 1).

### Blood glucose and insulin

**Figure 2:**
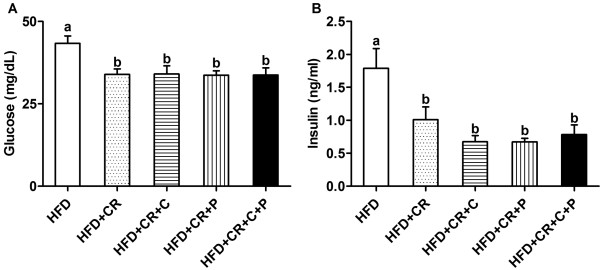
**Effect of CR and spice compounds on plasma glucose and insulin concentrations.** Plasma was collected and the glucose (**A**) and insulin (**B**) levels were determined by using Glucose Colorimetric Assay Kit and Ultra Sensitive Mouse Insulin ELISA Kit, respectively. Values are means ± SEM, n = 8 mice/group. Means without a common letter differ at *P < 0.05*. HFD: high fat diet, CR: caloric restriction, C: curcumin, P: piperine.

Compared to the HFD control group, the mice in all CR groups had lower plasma levels of glucose (Figure 2A) and insulin (Figure 2B). However, the addition of curcumin and/or piperine did not affect these CR-induced changes (Figure 2).

### Total spleen cell number and percent CD4^+^ T cells

**Figure 3:**
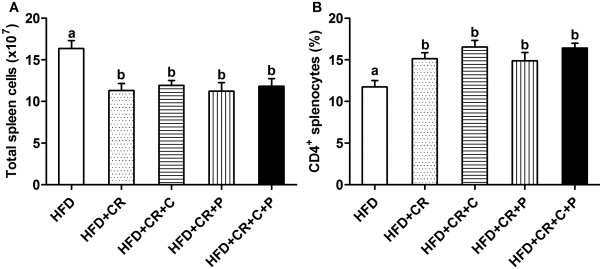
**Effect of CR and spice compounds on total number of spleen cells and proportion of CD4**^**+ **^**T cells. **Isolated splenocytes were counted under microscope for total spleen cells (**A**) and stained with fluorescence-conjugated anti-CD4 antibody, followed by flow cytometry to determine the splenic CD4^+ ^T cell levels (**B**). Values are means ± SEM, n = 9 mice/group. Means without a common letter differ at *P < 0.05*. HFD: high fat diet, CR: caloric restriction, C: curcumin, P: piperine.

Spleens from CR treated mice had lower total cell counts compared to the mice in HFD control group (Figure 3A), which reflected an overall smaller size of spleens (data not shown) as we reported before [24]. In agreement with a previous study [10], we also found that CR treated mice had a higher percentage of CD4^+^ T cells in total spleen cells (Figure 3B). However, the addition of curcumin and/or piperine did not affect these CR-induced changes (Figure 3).

### Production of pro-inflammatory cytokines by splenocytes

**Figure 4:**
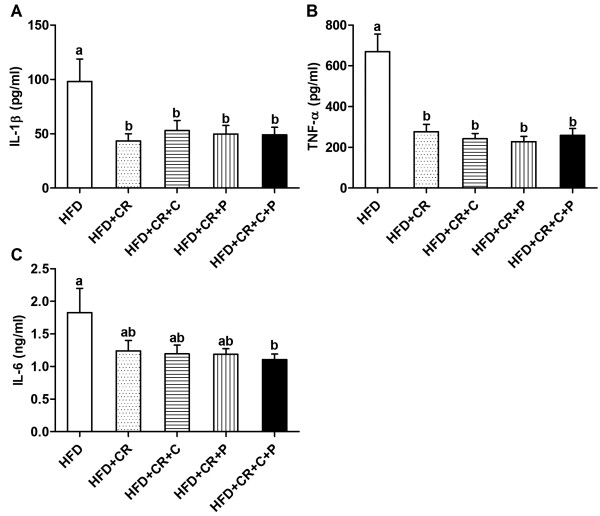
**Effect of CR and spice compounds on inflammatory cytokine production by splenocytes.** Spleen cells were stimulated with LPS for 24 h and supernatant was used to determine production of IL-1β (**A**), TNF-α (**B**), and IL-6 (**C**) using ELISA. Values are means ± SEM (n = 9 mice/group). Means without a common letter differ at *P < 0.05*. HFD: high fat diet, CR: caloric restriction, C: curcumin, P: piperine.

Splenocytes from CR treated mice produced less IL-1β and TNF-α compared to those from HFD control mice, and this anti-inflammatory effect of CR was not further potentiated by the concurrent supplementation with curcumin and/or piperine (Figure 4A and B). IL-6, another pro-inflammatory cytokine, also tended to be lower in all CR groups but in the curcumin + piperine group only, this decrease reached significance while in all the other CR groups, there was a strong trend toward decreases with varied p values at 0.06 to 0.08 (Figure 4C).

### Splenocyte proliferation

**Figure 5:**
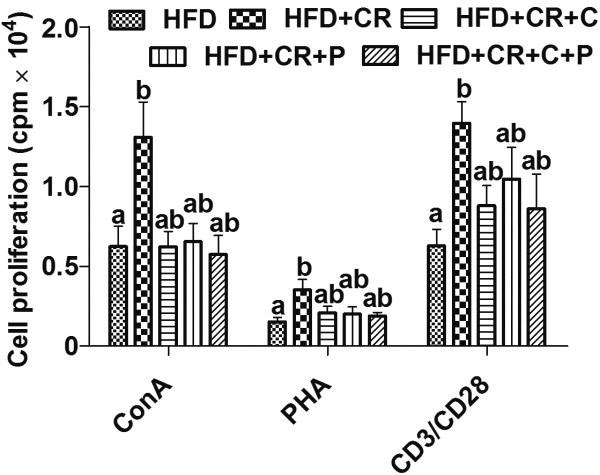
**Effect of CR and spice compounds on splenocyte proliferation. **Splenocytes were stimulated with T cell mitogen Con A or PHA, or anti-CD3/CD28 at their optimal concentrations (1.5, 5, 5/1 μg/ml, respectively) for 72 h, and proliferation were determined by [^3^H]-thymidine incorporation. Values are means ± SEM (n = 9 mice/group). Means without a common letter differ at *P < 0.05*. HFD: high fat diet, CR: caloric restriction, C: curcumin, P: piperine.

In the *ex vivo* cell proliferation experiments, we used different concentrations of T cell mitogens Con A and PHA, and anti-CD3/CD28. Since the effects of treatment on splenocyte proliferation was similar across concentrations used, we only showed the results generated with the optimal concentration of each stimulant used to induce T cell proliferation in splenocytes, i.e., Con A at 1.5 μg/ml, PHA at 5 μg/ml, and plate-coated anti-CD3 at 5 μg/ml plus soluble anti-CD28 at 1 μg/ml. We found that mice in only the CR group had higher levels of T cell proliferation in response to all the stimulation conditions compared to HFD control mice; however, this effect of CR was lessened to varied degrees by the concurrent curcumin and/or piperine supplementation so that proliferation levels in all the CR + curcumin and/or piperine groups did not differ from either HFD or CR group (Figure 5).

### Ex vivo production of T cell cytokines by splenocytes

**Figure 6:**
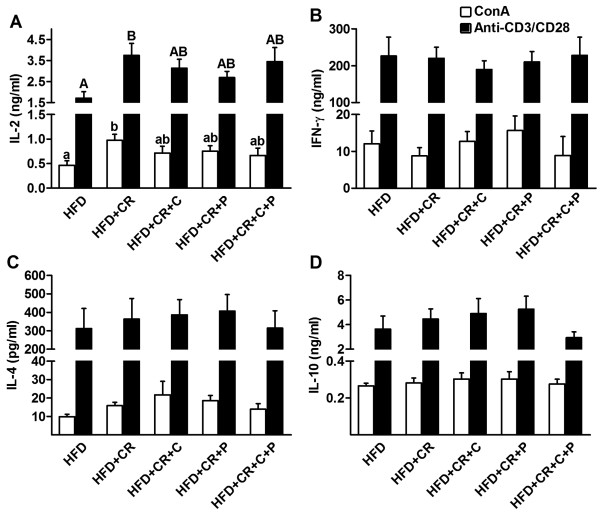
**Effect of CR and spice compounds on T cell cytokine production. **Splenocytes were stimulated with Con A (1.5 μg/ml) and plate-coated anti-CD3 (5 μg/ml)/anti-CD28 (1 μg/ml) for 48 h and supernatant was collected to determine production of T cell cytokines IL-2 (**A**), IFN-γ (**B**), IL-4 (**C**), IL-10 (**D**) using ELISA. Values are means ± SEM (n = 9 mice/group). Means without a common letter differ at *P < 0.05*. HFD: high fat diet, CR: caloric restriction, C: curcumin, P: piperine.

**Figure 7:**
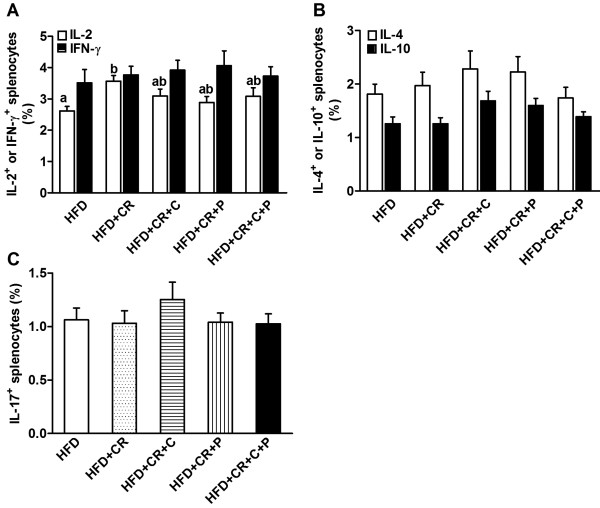
**Effect of CR and spice compounds on intracellular levels of T cell cytokines.** Splenocytes were stimulated with 50 ng/ml PMA and 500 ng/ml ionomycin in the presence of Golgi Stop for 4 h and intracellular IL-2 and IFN-γ (**A**), IL-4 and IL-10 (**B**), and IL-17 (**C**) were determined using flow cytometry. Values are means ± SEM (n = 9 mice/group). Means without a common letter differ at *P < 0.05*. HFD: high fat diet, CR: caloric restriction, C: curcumin, P: piperine.

Secretion of selected T cytokines was determined using ELISA in the cultures stimulated by either Con A or anti-CD3/CD28. Similar to the results of splenocyte proliferation, both Con A- and anti-CD3/CD28-stimulated splenocytes from mice in only the CR group produced more IL-2 compared to HFD control mice, and this effect was lessened by the concomitant curcumin and/or piperine supplementation (Figure 6A). No significant difference was found in splenocyte production of IFN-γ (Figure 6B), IL-4 (Figure 6C), and IL-10 (Figure 6D) across all groups. To determine if the observed change, or lack thereof, in cytokine production with CR was due to alteration in synthesis or post-secretion regulation of the cytokines, we determined the intracellular levels of these cytokines specifically in splenocytes using flow cytometry. As seen in Figures 7A and B, the results are generally in agreement with the above-mentioned observations. Furthermore, we determined intracellular levels of pro-inflammatory cytokine IL-17, the hallmark cytokine for Th17, and found no difference between different groups (Figure 7C).

### PGE_2_ production by splenocytes

**Figure 8:**
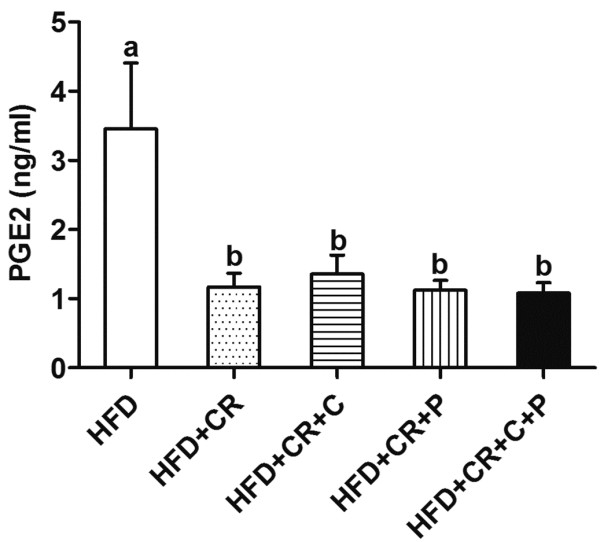
**Effect of CR and spice compounds on LPS-stimulated PGE**_**2 **_**production. **Spleen cells were stimulated with LPS for 24 h and supernatant was used to determine PGE_2 _production using a MSD kit and platform. Values are means ± SEM (n = 6 mice/group). Means without a common letter differ at *P < 0.05*. HFD: high fat diet, CR: caloric restriction, C: curcumin, P: piperine.

Consistent with our previous results generated in mice [24] and human subjects [7], we found that LPS-stimulated PGE_2_ production by splenocytes in CR mice was significantly lower than in HFD control mice. No further reduction in PGE_2_ production was observed by addition of curcumin and/or piperine (Figure 8).

### CD4^+^ T cell subpopulations in spleen cells

**Figure 9:**
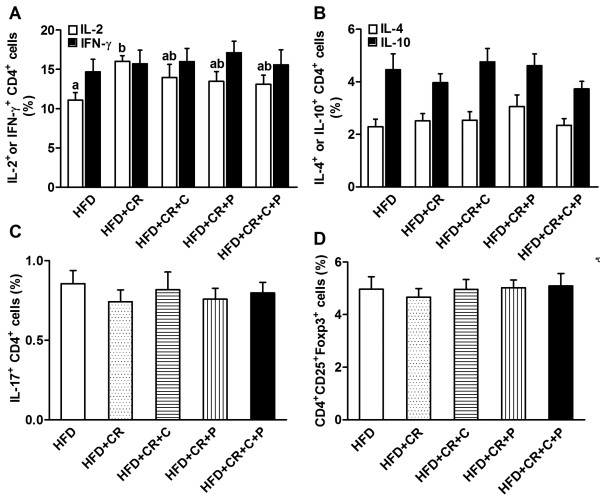
**Effect of CR and spice compounds on CD4**^**+ **^**T cell subpopulations. **Splenocytes were stimulated with 50 ng/ml PMA and 500 ng/ml ionomycin in the presence of Golgi Stop for 4 h. After that, appropriate surface and intracellular stainings were performed to identify CD4^+^ T cell subpopulations as defined by their cytokine production pattern: Th1 (IL-2 and IFN-γ) (**A**), Th2 (IL-4 and IL-10) (**B**), Th17 (IL-17) (**C**), and Treg (CD4^+^CD25^+^Foxp3^+^) (**D**) using flow cytometry. All analyses were performed within the gated CD4^+ ^T cells. Values are means ± SEM (n = 9 mice/group). Means without a common letter differ at *P < 0.05*. HFD: high fat diet, CR: caloric restriction, C: curcumin, P: piperine.

Increasing evidence points to the distinct roles of different CD4^+^ T cell subsets in anti-microbial immune response, allergies, tolerance, autoimmunity, and inflammation [25,26]. However, it is not known whether a combined CR and spice supplementation regimen would affect CD4^+^ T cell profiles as assessed by the production of their respective signature cytokines. We found that CR alone resulted in a higher production of IL-2 in CD4^+^ T cells, as indicated by the presence of more IL-2 producing CD4^+^ T cells, which was diminished when combined with spice supplementation (Figure 9A). CR and spice compounds, either alone or combined, had no effect on the proportion of CD4^+^ T cell subsets Th1 (IFN-γ), Th2 (IL-4, IL-10), Th17 (IL-17), and Treg cells (CD4^+^CD25^+^Foxp3^+^) (Figure 9).

## Discussion

It has been well-accepted that the high fat/high caloric content present in the typical Western diet adversely impacts health and is a common cause of obesity. In turn, studies have provided evidence linking obesity to the high risk of developing type 2 diabetes, cardiovascular disease, and metabolic syndrome. It is believed that a possible mechanism underlying this link is a chronic, low-grade inflammatory state developed as a consequence of increased adiposity, which has been supported by numerous studies in the past decade. While the obesity-associated inflammation has been extensively studied, the impact of obesity on the innate and adaptive immune response, in particular that of T cell-mediated function, has not been well-clarified thus far, though available evidence suggests that obesity may compromise the immune-surveillance pathways leading to impaired immune response against pathogens [27]. Further, the effect of weight reduction by CR and its combination with spice components on immune and inflammatory responses of obese mice has not been well-defined. Here we report that CR results in a reduction in body weight, fat mass, and inflammatory response, as well as an enhancement in T cell-mediated function compared to the mice fed the same high fat diet *ad libitum*. We also report that the addition of spice components curcumin and piperine does not further enhance CR’s benefit, but rather it blunts the effect of CR on T cell-mediated function.

In this study, we found that CR applied to the obese mice provided metabolic benefits as indicated by the reduced weight gain and fat mass and by lowered levels of fast blood glucose and insulin. These results agree with previous reports on CR in both humans [28,29] and animals [30,31]. Contrary to what we anticipated, concurrent consumption of curcumin and/or piperine had no further effect on these CR-induced changes. However, this is consistent with the similar lack of additional effects of these spice compounds on inflammation profile given the currently recognized association between adiposity, inflammatory state, and glucose metabolism.

Previous studies have reported that CR administered to lean or obese rodents increases total T cell population and both CD4^+^ and CD8^+^ subpopulations [10,32,33]. Consistent with these previous results, in the current study, which focused on CD4^+^ T cells and their subpopulations, we found that spleens from CR mice had higher percentages of CD4^+^ T cells. This increase in the proportion of CD4^+^ T cells may partly explain the higher levels of splenocyte proliferation in CR mice because only T cells in the spleen cell mixture should proliferate after being stimulated by T cell mitogens or TCR antibodies, and a larger number of T cells to begin with would be expected to result in a larger number of proliferating cells in the end. However, we cannot rule out that CR might have enhanced the T cell division and thus the proliferation rate.

Also consistent with the previous studies showing enhanced T cell proliferation and IL-2 production in CR mice and humans [7,33-35], we found that obese mice subjected to CR had higher levels of splenocyte proliferation and IL-2 production in response to both T cell mitogen and anti-CD3/CD28 stimulation. Of note, CR mice also had lower PGE_2_ production compared to the control mice, which confirmed our previous findings in mice and humans. Previously, we showed that 21% CR for 13 mo in Emory mice reduced PGE_2_ production in spleen [24]; in a recent human study, we found that 30% CR for 6 mo decreased PGE_2_ production in LPS-stimulated whole blood culture [7]. Given that PGE_2_ is a suppressive factor for T cell proliferation and IL-2 production [36-38], a reduction in PGE_2_ production by CR represents another mechanism through which CR might have improved T cell proliferation and IL-2 production in obese mice. We did not evaluate the mechanism of CR-induced decrease in PGE_2_ production in the current study. However, a study by Kim et al. provided insight into the underlying mechanism [39]. They reported that in kidneys from CR rats, the activity of cyclooxygenase (COX), a rate-limiting enzyme for eicosanoid synthesis, was lower compared to the control rats, and this change was accompanied by a lower expression of COX-2 at both protein and mRNA levels [39]. Although their findings for CR-induced changes were from different tissues (kidney vs. spleen in the current study), we speculate that the down-regulated COX-2 abundance/activity may represent a common mechanism shared across different tissues for the observed effect of CR on PGE_2_ production. CR’s inhibitory effect on PGE_2_ production also provided another piece of evidence to support the suggested anti-inflammatory property of CR given the fact that PGE_2_ is a very potent lipid inflammatory mediator and it is involved in the pathogenesis of many inflammatory diseases.

In this study, we found that CR’s enhancing effect on T cell proliferation and IL-2 production was lessened by supplementation of CR diets with spice compounds to such a level that CR’s effect was no longer significant in any of the CR groups that were supplemented with curcumin and/or piperine. This may reflect a suppressive effect of curcumin and piperine on T cell-mediated functions as reported in several previous studies [40-44]. For example, it has been shown that dietary curcumin in mice suppresses CD4^+^ T cell proliferation and IL-2 production [43] while *in vitro* curcumin supplementation inhibits T cell activation [44], human peripheral blood mononuclear cell proliferation and IL-2 production [40,42], and IL-2 synthesis as well as IL-2 signaling in mouse CD4+ T cells [41]. Although little information is available for piperine in this regard, some studies have indeed suggested that piperine may inhibit T cell-related functions [19,45].

Both CR and curcumin have been shown to have an anti-inflammatory effect, respectively. Therefore, in this study, we investigated whether inclusion of curcumin in a CR diet would further potentiate CR’s beneficial effects. While our results clearly showed an anti-inflammatory effect of CR, which was indicated by a significant reduction of IL-1β and TNF-α as well as a strong trend toward some reduction of IL-6, curcumin and piperine demonstrated no additional benefits. These results lend further support to the suggested beneficial effects of CR toward mitigating inflammation caused by an HFD/obesity condition. Of note, it was a study limitation that all spice compound-supplemented mice were on CR treatment; therefore, it is presently unknown whether these bioactive compounds would have produced an anti-inflammatory effect if administered to HFD control mice without CR regimen.

An increased presence of immune cells in adipose tissue is the hallmark of obesity-induced inflammation. Macrophages were first found in the adipose tissue of obese animals and humans nearly a decade ago [46,47]. Emerging evidence now suggests that during obesity, T cells precede macrophages in entering adipose tissue and produce pro-inflammatory cytokines to activate macrophages. It is still unresolved as to which of the CD4^+^ and CD8^+^ T cells are the predominant T cells present in the adipose tissue of obese humans and animals and how obesity affects the balance within CD4^+^ T cells and among their different subpopulations, i.e., Th1, Th2, Th17, Treg, etc. The qualitative and quantitative changes in the immune cells present in adipose tissue are proposed to be key factors in the obesity-inflammation-metabolic disorder pathway. In particular, different CD4^+^ T cell subsets have recently attracted increasing attention due to the fact that specific types of CD4^+^ T cells are known to play different roles in maintaining homeostasis as well as regulating immune and inflammatory responses [25,26]. In contrast to our study’s hypothesis, we determined that CR and spice compounds, alone or in combination, have no effect on CD4^+^ subpopulations in peripheral lymphoid tissue (spleen). This indicates that neither CR nor the spice compounds affect CD4^+^ T cell differentiation under HFD/obesity. However, it remains possible that CR may favorably affect CD4^+^ T cell differentiation under certain other conditions. In a recent study [48], CR in fact attenuated experimental autoimmune encephalomyelitis (EAE, a rodent model for multiple sclerosis) in mice, an autoimmune disease driven by antigen-specific Th1 and Th17 response and mitigated by Treg cells; however, CD4^+^ T cell subsets were not examined in that study. Information regarding the impact of spices on CD4 subpopulation is limited. Dietary curcumin was shown to ameliorate EAE in rats through inhibition of Th17 response [49] and in mice through inhibition of Th1/Th17 responses and enhancement of Th2/Treg responses [50]. Taken together, these results indicate a potential area for future studies to explore: whether the beneficial effect of CR on autoimmune diseases is mediated through its effect on CD4^+^ T cell differentiation and further, whether CR interacts with spice compound supplementation to mutually potentiate their respective effectiveness.

## Conclusions

We have demonstrated that CR has a beneficial effect in diet-induced obesity by suppressing the inflammatory response and improving T cell-mediated function in obese mice. This effect of CR on T cell mediated function might be due in part to an increase in CD4^+^ cells and a reduction in PGE_2_ production. Contrary to our hypothesis, however, supplementation of CR diet with bioactive spice compounds curcumin and/or piperine provides no additional benefit; instead, they diminished CR’s effect on T cell-mediated function. Future studies are needed to further determine the mechanisms underlying these observations as well as the applicability of these findings to humans.

## Abbreviations

CR: Caloric restriction; ConA: Concanavalin A; HFD: High-fat diet; LPS: Lipopolysaccharide; PGE2: Prostaglandin E_2_; PHA: Phytohemagglutinin; Treg: Regulatory T cells; Th: Helper T cells.

## Competing interests

The authors declare that they have no competing interests.

## Authors’ contributions

MM, SNM, DW, and JW participated in the study design, data interpretation, and manuscript writing. JW performed all immunological assays and statistical analyses. SV and XD helped in sample collection, immune cell isolation, and cytokine analysis. JAZ and TN carried out animal feeding and care, body weight data collection and analysis, and helped in manuscript revision. All authors read and approved the final manuscript.

## Authors’ information

Mohsen Meydani, Simin Nikbin Meydani and Dayong Wu share senior authorship.
